# (*E*)-4-Chloro-2-{[4-(3,5-dichloro­pyridin-2-yl­oxy)phenyl­imino]­meth­yl}phenol

**DOI:** 10.1107/S1600536811041584

**Published:** 2011-10-22

**Authors:** Hong-Bo Ji, Pei-Zhi Zhang, Jun Wu

**Affiliations:** a Department of Chemistry, Zhejiang University, Hangzhou 310027, People’s Republic of China; b School of Biological and Chemical Engineering, Zhejiang University of Science and Technology, Hangzhou 310012, People’s Republic of China

## Abstract

In the title mol­ecule, C_18_H_11_Cl_3_N_2_O_2_, the central benzene ring is oriented at 8.44 (12) and 70.57 (11)° with respect to the terminal chloro­phenol and dichloro­pyridine rings, respectively. The mol­ecular structure is stabilized by an intra­molecular O—H⋯N hydrogen bond, which generates an *S*(6) ring motif. In the crystal, π–π stacking between parallel pyridine rings is observed [centroid–centroid distance = 3.6561 (14) Å].

## Related literature

For general background to the pharmacological activity of Schiff base compounds, see: Shapiro (1998 ▶); Venugopal & Jayashree (2008 ▶); Pandey *et al.* (2003 ▶); Bhat *et al.* (2005 ▶); Wadher *et al.* (2009 ▶). For a related structure, see: Fun *et al.* (2011 ▶).
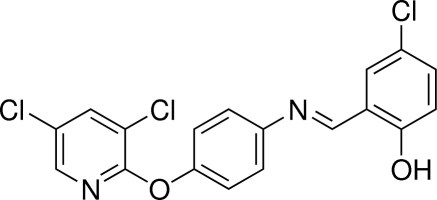


## Experimental

### 

#### Crystal data

C_18_H_11_Cl_3_N_2_O_2_
*M* *_r_* = 393.64
Monoclinic, 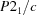

*a* = 13.8981 (7) Å
*b* = 11.7006 (8) Å
*c* = 10.5034 (5) Å
β = 95.318 (5)°
*V* = 1700.67 (16) Å^3^
*Z* = 4
Mo *K*α radiation
μ = 0.55 mm^−1^
*T* = 293 K
0.38 × 0.36 × 0.29 mm

#### Data collection

Oxford Diffraction Xcalibur Atlas Gemini ultra diffractometer
Absorption correction: multi-scan (*CrysAlis PRO*; Oxford Diffraction, 2010 ▶) *T*_min_ = 0.817, *T*_max_ = 0.856
7882 measured reflections
3107 independent reflections
2186 reflections with *I* > 2σ(*I*)
*R*_int_ = 0.025

#### Refinement

*R*[*F* ^2^ > 2σ(*F* ^2^)] = 0.040
*wR*(*F* ^2^) = 0.100
*S* = 1.03
3107 reflections
227 parameters
H-atom parameters constrained
Δρ_max_ = 0.27 e Å^−3^
Δρ_min_ = −0.26 e Å^−3^

### 

Data collection: *CrysAlis PRO* (Oxford Diffraction, 2010 ▶); cell refinement: *CrysAlis PRO*; data reduction: *CrysAlis PRO*; program(s) used to solve structure: *SHELXS97* (Sheldrick, 2008 ▶); program(s) used to refine structure: *SHELXL97* (Sheldrick, 2008 ▶); molecular graphics: *OLEX2* (Dolomanov *et al.*, 2009 ▶); software used to prepare material for publication: *OLEX2*.

## Supplementary Material

Crystal structure: contains datablock(s) I, global. DOI: 10.1107/S1600536811041584/xu5335sup1.cif

Structure factors: contains datablock(s) I. DOI: 10.1107/S1600536811041584/xu5335Isup2.hkl

Supplementary material file. DOI: 10.1107/S1600536811041584/xu5335Isup3.cml

Additional supplementary materials:  crystallographic information; 3D view; checkCIF report

## Figures and Tables

**Table 1:** Hydrogen-bond geometry (Å, °)

*D*—H⋯*A*	*D*—H	H⋯*A*	*D*⋯*A*	*D*—H⋯*A*
O1—H1⋯N1	0.82	1.86	2.586 (3)	147

**Fig. 1.**
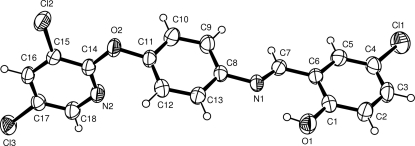
The molecular structure of title compound, with atom labels and 50% probability displacement ellipsoids for non-H atoms.

## supplementary crystallographic information

### Comment

In 2011, much attention has been focused on the biological properties of Schiff bases compounds, Schiff base ligands may contain a variety of substituents with different electron-donating or electron-withdrawing groups and therefore may have interesting chemical properties. They have attracted particular interest due to their biological activities (Shapiro, 1998). They have been found to posses the pharmacological activities such as antimalarial, anticancer, antibacterial (Venugopal & Jayashree, 2008), antifungal (Pandey *et al.*, 2003), antitubercular (Bhat *et al.*, 2005), anti-inflammatoryand antimicrobial (Wadher *et al.*, 2009) properties.

The crystal structures of a number of Schiff bases compounds have also been determined. Herein, we report on the synthesis and crystal structure of a new Schiff bases compound, prepared by the reaction of 5-chloro-2-hydroxybenzaldehyde with 4-(3,5-dichloropyridin-2-yloxy) benzenamine.

In the title molecule (Fig. 1), the C8-benzene ring forms dihedral angles of 8.5 (2)° and 70.6 (1)° with the chlorophenol and dichloropyridine rings, respectively. The title molecule exists in *trans* configuration with respect to the C7=N1 bond [C7=N1 = 1.275 (3) Å]. In the crystal packing, π-π stacking interactions between the centroid of C1—C6 (*Cg*1) and C8—C13 (*Cg*2) benzene rings, with *Cg*1···*Cg*2^i^ distance of 3.792 (2) Å [symmetry code: (i) -*x*, 1 - *y*, 2 - *z*] are observed. N2/C14—C18 (*Cg*3)···*Cg*3^ii^ distance of 3.656 (1)Å [symmetry code: (ii), 1 - *x*,1 - *y*,1 - *z*]. The molecular structure is stabilized by an intramolecular O–H···N hydrogen bond, which generates an S(6) ring motif (Table 1). No significant intermolecular hydrogen bonds are observed.

### Experimental

The title compound was prepared by the condensation reaction of 5-chloro-2-hydroxybenzaldehyde (5 mmol, 0.78 g) and 4-(3,5-dichloropyridin-2-yloxy)benzenamine (5 mmol, 1.27 g) in anhydrous methanol (30 ml) at ambient temperature. The solution was magnetically stirred at ambient temperature for 10 min until it turned to yellow. Yellow single crystals suitable for X-ray structural determination were obtained by slow evaporation of the solution for 7 d.

### Refinement

H atoms were placed in idealized positions (C—H = 0.93 Å, O—H= 0.82 Å), and refined as riding, with *U*_iso_(H) = 1.2*U*_eq_(C) and 1.5*U*_eq_(O).

### Crystal data

**Table:**

C_18_H_11_Cl_3_N_2_O_2_	*F*(000) = 800
*M**_r_* = 393.64	*D*_x_ = 1.537 Mg m^−^^3^
Monoclinic, *P*2_1_/*c*	Mo *K*α radiation, λ = 0.71073 Å
Hall symbol: -P 2ybc	Cell parameters from 1892 reflections
*a* = 13.8981 (7) Å	θ = 2.9–29.5°
*b* = 11.7006 (8) Å	µ = 0.55 mm^−^^1^
*c* = 10.5034 (5) Å	*T* = 293 K
β = 95.318 (5)°	Block, yellow
*V* = 1700.67 (16) Å^3^	0.38 × 0.36 × 0.29 mm
*Z* = 4	

### Data collection

**Table:**

Oxford Diffraction Xcalibur Atlas Gemini ultra diffractometer	3107 independent reflections
Radiation source: fine-focus sealed tube	2186 reflections with *I* > 2σ(*I*)
graphite	*R*_int_ = 0.025
Detector resolution: 10.3592 pixels mm^-1^	θ_max_ = 25.4°, θ_min_ = 2.9°
ω scans	*h* = −16→16
Absorption correction: multi-scan (*CrysAlis PRO*; Oxford Diffraction, 2010)	*k* = −14→8
*T*_min_ = 0.817, *T*_max_ = 0.856	*l* = −12→12
7882 measured reflections	

### Refinement

**Table:**

Refinement on *F*^2^	Primary atom site location: structure-invariant direct methods
Least-squares matrix: full	Secondary atom site location: difference Fourier map
*R*[*F*^2^ > 2σ(*F*^2^)] = 0.040	Hydrogen site location: inferred from neighbouring sites
*wR*(*F*^2^) = 0.100	H-atom parameters constrained
*S* = 1.03	*w* = 1/[σ^2^(*F*_o_^2^) + (0.0376*P*)^2^ + 0.5849*P*] where *P* = (*F*_o_^2^ + 2*F*_c_^2^)/3
3107 reflections	(Δ/σ)_max_ < 0.001
227 parameters	Δρ_max_ = 0.27 e Å^−^^3^
0 restraints	Δρ_min_ = −0.26 e Å^−^^3^

### Special details

**Table:**

Geometry. All e.s.d.'s (except the e.s.d. in the dihedral angle between two l.s. planes) are estimated using the full covariance matrix. The cell e.s.d.'s are taken into account individually in the estimation of e.s.d.'s in distances, angles and torsion angles; correlations between e.s.d.'s in cell parameters are only used when they are defined by crystal symmetry. An approximate (isotropic) treatment of cell e.s.d.'s is used for estimating e.s.d.'s involving l.s. planes.
Refinement. Refinement of *F*^2^ against ALL reflections. The weighted *R*-factor *wR* and goodness of fit *S* are based on *F*^2^, conventional *R*-factors *R* are based on *F*, with *F* set to zero for negative *F*^2^. The threshold expression of *F*^2^ > σ(*F*^2^) is used only for calculating *R*-factors(gt) *etc*. and is not relevant to the choice of reflections for refinement. *R*-factors based on *F*^2^ are statistically about twice as large as those based on *F*, and *R*- factors based on ALL data will be even larger.

### Fractional atomic coordinates and isotropic or equivalent isotropic displacement parameters (Å^2^)

**Table:**

	*x*	*y*	*z*	*U*_iso_*/*U*_eq_	
Cl1	−0.24721 (6)	0.26343 (9)	1.31374 (10)	0.1009 (3)	
Cl2	0.53771 (6)	0.21299 (7)	0.55024 (6)	0.0716 (2)	
Cl3	0.41754 (6)	0.53738 (7)	0.20843 (6)	0.0723 (2)	
O1	0.08837 (14)	0.54210 (18)	1.22171 (19)	0.0727 (6)	
H1	0.1140	0.5196	1.1591	0.109*	
O2	0.37377 (13)	0.29274 (16)	0.67705 (16)	0.0629 (5)	
N1	0.11798 (14)	0.40766 (18)	1.03385 (17)	0.0464 (5)	
N2	0.32327 (15)	0.4354 (2)	0.53329 (19)	0.0570 (6)	
C1	0.01103 (17)	0.4760 (2)	1.2388 (2)	0.0503 (6)	
C2	−0.0441 (2)	0.5020 (3)	1.3378 (2)	0.0618 (7)	
H2	−0.0275	0.5646	1.3899	0.074*	
C3	−0.1226 (2)	0.4374 (3)	1.3606 (3)	0.0635 (8)	
H3	−0.1592	0.4560	1.4274	0.076*	
C4	−0.14696 (18)	0.3448 (3)	1.2841 (3)	0.0605 (7)	
C5	−0.09479 (18)	0.3184 (2)	1.1840 (2)	0.0556 (6)	
H5	−0.1130	0.2563	1.1320	0.067*	
C6	−0.01468 (16)	0.3832 (2)	1.1588 (2)	0.0452 (6)	
C7	0.04132 (17)	0.3533 (2)	1.0541 (2)	0.0479 (6)	
H7	0.0209	0.2932	1.0004	0.057*	
C8	0.17777 (16)	0.3761 (2)	0.9376 (2)	0.0439 (6)	
C9	0.16860 (18)	0.2763 (2)	0.8667 (2)	0.0587 (7)	
H9	0.1192	0.2251	0.8796	0.070*	
C10	0.23239 (19)	0.2524 (3)	0.7770 (2)	0.0580 (7)	
H10	0.2258	0.1856	0.7290	0.070*	
C11	0.30536 (18)	0.3275 (2)	0.7592 (2)	0.0506 (6)	
C12	0.31653 (19)	0.4262 (2)	0.8286 (3)	0.0605 (7)	
H12	0.3665	0.4766	0.8159	0.073*	
C13	0.25235 (18)	0.4498 (2)	0.9179 (2)	0.0545 (6)	
H13	0.2596	0.5167	0.9657	0.065*	
C14	0.38386 (18)	0.3526 (2)	0.5682 (2)	0.0483 (6)	
C15	0.45916 (17)	0.3205 (2)	0.4973 (2)	0.0463 (6)	
C16	0.47057 (17)	0.3767 (2)	0.3851 (2)	0.0475 (6)	
H16	0.5199	0.3571	0.3351	0.057*	
C17	0.40660 (18)	0.4631 (2)	0.3490 (2)	0.0495 (6)	
C18	0.33466 (19)	0.4902 (2)	0.4238 (2)	0.0580 (7)	
H18	0.2921	0.5488	0.3978	0.070*	

### Atomic displacement parameters (Å^2^)

**Table:**

	*U*^11^	*U*^22^	*U*^33^	*U*^12^	*U*^13^	*U*^23^
Cl1	0.0704 (5)	0.0995 (7)	0.1413 (8)	−0.0127 (5)	0.0547 (5)	0.0032 (6)
Cl2	0.0835 (5)	0.0768 (5)	0.0579 (4)	0.0333 (4)	0.0250 (3)	0.0073 (4)
Cl3	0.0867 (5)	0.0815 (6)	0.0517 (4)	0.0132 (4)	0.0228 (3)	0.0158 (3)
O1	0.0733 (13)	0.0749 (15)	0.0738 (13)	−0.0208 (11)	0.0273 (10)	−0.0201 (11)
O2	0.0720 (12)	0.0679 (13)	0.0535 (10)	0.0212 (10)	0.0307 (9)	0.0124 (9)
N1	0.0459 (11)	0.0541 (13)	0.0406 (10)	0.0016 (10)	0.0124 (9)	0.0025 (9)
N2	0.0572 (13)	0.0677 (15)	0.0487 (12)	0.0133 (11)	0.0180 (10)	0.0065 (11)
C1	0.0479 (14)	0.0581 (17)	0.0461 (13)	−0.0003 (12)	0.0102 (11)	0.0045 (12)
C2	0.0675 (18)	0.0693 (19)	0.0505 (15)	0.0029 (15)	0.0158 (13)	−0.0090 (14)
C3	0.0595 (17)	0.080 (2)	0.0543 (15)	0.0136 (15)	0.0251 (13)	0.0055 (15)
C4	0.0474 (14)	0.0638 (19)	0.0734 (18)	0.0041 (13)	0.0226 (13)	0.0107 (15)
C5	0.0516 (14)	0.0543 (16)	0.0623 (16)	0.0013 (13)	0.0120 (13)	−0.0016 (13)
C6	0.0448 (13)	0.0490 (15)	0.0426 (12)	0.0053 (11)	0.0082 (10)	0.0051 (11)
C7	0.0503 (14)	0.0495 (15)	0.0445 (13)	0.0045 (12)	0.0079 (11)	−0.0029 (11)
C8	0.0442 (13)	0.0513 (15)	0.0372 (12)	0.0039 (11)	0.0083 (10)	0.0028 (11)
C9	0.0523 (15)	0.0645 (19)	0.0617 (16)	−0.0102 (13)	0.0188 (13)	−0.0100 (14)
C10	0.0607 (16)	0.0637 (18)	0.0505 (15)	0.0014 (14)	0.0097 (12)	−0.0125 (13)
C11	0.0549 (15)	0.0577 (17)	0.0415 (13)	0.0128 (13)	0.0164 (11)	0.0074 (12)
C12	0.0572 (15)	0.0607 (19)	0.0674 (17)	−0.0055 (13)	0.0259 (13)	−0.0009 (14)
C13	0.0580 (15)	0.0552 (17)	0.0523 (14)	−0.0028 (13)	0.0157 (12)	−0.0065 (12)
C14	0.0541 (14)	0.0533 (16)	0.0393 (13)	0.0034 (12)	0.0139 (11)	−0.0016 (11)
C15	0.0499 (13)	0.0476 (15)	0.0426 (12)	0.0033 (11)	0.0104 (11)	−0.0053 (11)
C16	0.0478 (13)	0.0570 (16)	0.0398 (12)	−0.0020 (12)	0.0149 (11)	−0.0093 (11)
C17	0.0555 (15)	0.0561 (16)	0.0377 (12)	−0.0022 (12)	0.0080 (11)	−0.0001 (11)
C18	0.0603 (16)	0.0653 (18)	0.0499 (15)	0.0129 (14)	0.0129 (13)	0.0074 (13)

### Geometric parameters (Å, °)

**Table:**

Cl1—C4	1.739 (3)	C5—H5	0.9300
Cl2—C15	1.724 (2)	C6—C7	1.448 (3)
Cl3—C17	1.732 (2)	C7—H7	0.9300
O1—C1	1.349 (3)	C8—C13	1.379 (3)
O1—H1	0.8200	C8—C9	1.384 (3)
O2—C14	1.359 (3)	C9—C10	1.381 (3)
O2—C11	1.402 (3)	C9—H9	0.9300
N1—C7	1.275 (3)	C10—C11	1.368 (4)
N1—C8	1.416 (3)	C10—H10	0.9300
N2—C14	1.314 (3)	C11—C12	1.367 (4)
N2—C18	1.339 (3)	C12—C13	1.381 (3)
C1—C2	1.381 (3)	C12—H12	0.9300
C1—C6	1.400 (3)	C13—H13	0.9300
C2—C3	1.366 (4)	C14—C15	1.391 (3)
C2—H2	0.9300	C15—C16	1.371 (3)
C3—C4	1.372 (4)	C16—C17	1.376 (3)
C3—H3	0.9300	C16—H16	0.9300
C4—C5	1.366 (3)	C17—C18	1.365 (3)
C5—C6	1.393 (3)	C18—H18	0.9300
			
C1—O1—H1	109.5	C10—C9—H9	119.8
C14—O2—C11	119.80 (19)	C8—C9—H9	119.8
C7—N1—C8	122.9 (2)	C11—C10—C9	119.6 (3)
C14—N2—C18	118.0 (2)	C11—C10—H10	120.2
O1—C1—C2	118.6 (2)	C9—C10—H10	120.2
O1—C1—C6	121.8 (2)	C12—C11—C10	121.2 (2)
C2—C1—C6	119.6 (2)	C12—C11—O2	121.6 (2)
C3—C2—C1	121.1 (3)	C10—C11—O2	116.9 (2)
C3—C2—H2	119.4	C11—C12—C13	118.9 (2)
C1—C2—H2	119.4	C11—C12—H12	120.6
C2—C3—C4	119.5 (3)	C13—C12—H12	120.6
C2—C3—H3	120.3	C8—C13—C12	121.2 (2)
C4—C3—H3	120.3	C8—C13—H13	119.4
C5—C4—C3	120.6 (3)	C12—C13—H13	119.4
C5—C4—Cl1	120.3 (2)	N2—C14—O2	120.1 (2)
C3—C4—Cl1	119.0 (2)	N2—C14—C15	123.0 (2)
C4—C5—C6	120.9 (3)	O2—C14—C15	116.9 (2)
C4—C5—H5	119.5	C16—C15—C14	118.9 (2)
C6—C5—H5	119.5	C16—C15—Cl2	120.45 (18)
C5—C6—C1	118.2 (2)	C14—C15—Cl2	120.63 (18)
C5—C6—C7	120.6 (2)	C15—C16—C17	117.8 (2)
C1—C6—C7	121.2 (2)	C15—C16—H16	121.1
N1—C7—C6	121.5 (2)	C17—C16—H16	121.1
N1—C7—H7	119.2	C18—C17—C16	120.0 (2)
C6—C7—H7	119.2	C18—C17—Cl3	120.1 (2)
C13—C8—C9	118.7 (2)	C16—C17—Cl3	119.89 (19)
C13—C8—N1	116.3 (2)	N2—C18—C17	122.3 (2)
C9—C8—N1	125.0 (2)	N2—C18—H18	118.8
C10—C9—C8	120.3 (2)	C17—C18—H18	118.8

### Hydrogen-bond geometry (Å, °)

**Table:**

*D*—H···*A*	*D*—H	H···*A*	*D*···*A*	*D*—H···*A*
O1—H1···N1	0.82	1.86	2.586 (3)	147.
